# Identification of an Immature Subset of PMN-MDSC Correlated to Response to Checkpoint Inhibitor Therapy in Patients with Metastatic Melanoma

**DOI:** 10.3390/cancers13061362

**Published:** 2021-03-17

**Authors:** Françoise Gondois-Rey, Magali Paul, Florence Alcaraz, Sarah Bourass, Jilliana Monnier, Nausicaa Malissen, Jean-Jacques Grob, Annika M. Bruger, Pierre Van Der Bruggen, Caroline Gaudy-Marqueste, Daniel Olive

**Affiliations:** 1 Immunity and Cancer Team, Centre de Recherche en Cancérologie de Marseille (CRCM), Inserm, U1068, CNRS, UMR7258, Institut Paoli-Calmettes, Aix Marseille University, UM105, 13009 Marseille, France; magali.paul@imcheck.fr (M.P.); florence.alcaraz@imcheck.fr (F.A.); sarah.bourass@imcheck.fr (S.B.); jean-jacques.grob@ap-hm.fr (J.-J.G.); caroline.gaudy@ap-hm.fr (C.G.-M.); 2 Service de Dermatologie et de Cancérologie Cutanée, Hôpital de la Timone, 13005 Marseille, France; jilliana.monnier@ap-hm.fr (J.M.); nausicaa.malissen@ap-hm.fr (N.M.); 3 De Duve Institute, Université Catholique de Louvain, 1200 Brussels, Belgium; annika.bruger@uclouvain.be (A.M.B.); pierre.vanderbruggen@uclouvain.be (P.V.D.B.); 4 Walloon Excellence in Life Sciences and BIOtechnology (WELBIO), 1200 Brussels, Belgium

**Keywords:** PMN-MDSC, immune suppression, metastatic melanoma, immune-checkpoint therapy, signal regulatory protein alpha

## Abstract

**Simple Summary:**

Polymorphonuclear myeloid-derived suppressive cells (PMN-MDSCs) have been associated to bad prognosis and resistance to immune checkpoint inhibitor (ICI) therapy in metastatic melanoma (MM). In this study, we describe an immature subset of PMN-MDSCs capable of a high cytotoxicity against T cells mediated by a MAC-1 interaction, characterized by the absence of expression of the signal regulatory protein alpha. This subset is increased in patients responding to ICI therapy. Although the processes involving these cells in vivo are unknown, low-density CD15^+^SIRPα^−^ cells might constitute a useful biomarker to monitor clinical response in MM patients.

**Abstract:**

PMN-MDSCs support tumor progression and resistance to ICI therapy through their suppressive functions but their heterogeneity limits their use as biomarkers in cancer. Our aim was to investigate the phenotypic and functional subsets of PMN-MDSCs to identify biomarkers of response to ICI therapy. We isolated low-density CD15^+^ PMNs from patients with metastatic melanoma and assessed their immune-suppressive capacities. Expression of CD10 and CD16 was used to identify mature and immature subsets and correlate them to inhibition of T cell proliferation or direct cytotoxicity. Frequencies of the PMN-MDSCs subsets were next correlated to the radiological response of 36 patients receiving ICI therapy. Mature activated cells constituted the major population of PMN-MDSCs. They were found in a higher proportion in the pre-treatment blood of patients non responders to ICI. A subset of immature cells characterized by intermediate levels of CD10 and CD16, the absence of expression of SIRPα and a strong direct cytotoxicity to T cells was increased in patients responding to ICI. The paradoxical expansion of such cells during ICI therapy suggests a role of PMNs in the inflammatory events associated to efficient ICI therapy and the usefulness of their monitoring in patients care.

## 1. Introduction

Myeloid-derived suppressive cells (MDSCs) have become increasingly attractive for the development of immune-based therapies against cancer, as both potential bio-markers to monitor treatment response and targets to counteract immune suppression and resistance to treatment. MDSCs emerge from myeloid lineages under pathological conditions [1,2] and comprise cells derived from the monocytic (M-MDSC) lineage, the granulocytic polymorphonuclear (PMN-MDSC) lineage or more immature myeloid cells (early-MDSC) [3]. In metastatic melanoma, high MDSC frequencies in the blood correlate with poor prognosis [4,5,6] and MDSC numbers decrease after response to ICI [7,8].

PMN-MDSCs are commonly defined by their low-density in Ficoll gradients while normal PMNs are separated from peripheral blood mononuclear cells (PBMCs) by their high-density. Low-density PMNs are expanded in cancer [9,10] and their rapid decrease under efficient therapy suggest that they may constitute interesting biomarkers [7]. Low-density PMNs share many attributes with high-density PMNs including high granularity and the expression of granulocyte surface markers [11]. They are heterogeneous and contain populations expressing combinations of markers that suggest various stages of maturation or activation [12]. However, low cell density is not a feature specific to PMN-MDSCs as the density of PMNs decreases upon in vitro activation [9]. Cells isolated as low-density PMN-MDSCs could thus include activated PMNs, which are commonly expanded in cancer [13].

MDSCs are principally defined by their suppressive capacities [14]. PMN-MDSCs can use cytotoxicity and phagocytosis, two characteristic functions of their lineage. Cytotoxicity results from the release of toxic granules accumulated during maturation and of ROS produced upon activation of the NADPH oxidase in the “respiratory burst” [15]. Trogocytosis, the mechanism of phagocytosis employed by PMNs, is characterized by a mechanic destruction of the plasma membrane inducing cell death by lytic processes and necrosis [16,17]. This mechanism was demonstrated in PMNs’ ADCC (antibody-dependent-cell-cytotoxicity) against tumor cells opsonized with therapeutic antibodies [17,18]. Like phagocytosis, trogocytosis requires adhesion mediated by the β2-integrin MAC-1. Upon activation, MAC-1 undergoes rapid conformational changes that allows the formation of a synapse with the target cell [18]. Phagocytosis is controlled by the “don’t eat me” axis constituted by the interaction of the signal-regulatory-protein-alpha (SIRPα), a myeloid checkpoint, with its counter-receptor CD47, a self-molecule widely expressed on healthy cells [19]. Inhibition of MAC-1 is one of the mechanisms triggered by the interaction of SIRPα with CD47 [20].

Intra-cellular expression of arginase-1 (ARG1) [21,22] or reactive oxygen species (ROS) [9,23] have been found in PMN-MDSCs, however, assays showing direct suppression of T cell activity are recommended to assess the suppressive function [3,24]. Although normal high-density PMNs are not supposed to suppress T cells, they were shown to develop a suppressive capacity resembling that of low-density PMN-MDSCs as a result of uncontrolled stimulation during their separation from blood or after stimulation [9,25,26].

The immunosuppressive capacity of PMN-MDSCs is usually assessed by the inhibition of T cell proliferation or cytokine release evaluated after stimulation by cross-linking of CD3/CD28 and a few days of co-culture. In such assays, high suppressive activity has been attributed to either CD10-expressing mature [27,28], or LOX-1-expressing PMN-MDSCs. LOX-1 is a class E scavenger receptor reportedly specific to suppressive PMN-MDSCs [29] and recent studies showed a spatial accumulation of infiltrating LOX-1^+^ PMNs with inactivated cytotoxic T cells suggesting these cells contribute to tumor progression by suppressing the functions of infiltrating T cell [30]. Low-density PMNs are heterogeneous and may also contain immature cells with immune stimulatory functions, as those described in patients in whom inflammation was induced by G-CSF [31].

Although direct cytotoxicity is the primary process used by PMNs [15], immune suppression of the low density PMN-MDSCs found in cancer patients in this form has not been studied. In an attempt to identify relevant biomarkers of response to ICI we analyzed a cohort of patients with metastatic melanoma undergoing ICI therapy and we characterized low-density CD15^+^ PMNs by their ability to inhibit T cell proliferation or directly kill T cells and by their maturity phenotype.

## 2. Results

### 2.1. Low-Density CD10^+^ PMNs and High-Density PMNs Similarly Suppress T Cell Proliferation

**Figure 1:**
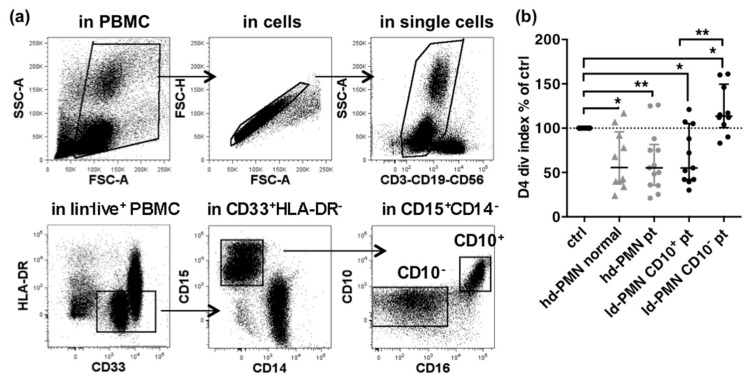
Suppression of T cells proliferation by high-density PMNs or PMN-MDSCs. (**a**) Sorting of PMN-MDSCs in PBMC. MDSCs are gated as CD33^+^HLA-DR^−^ cells within live CD3^–^CD19^–^CD56^–^ (lin^−^) cells after exclusion of debris, doublets and dead cells. PMN-MDSCs are gated as CD14^–^CD15^+^ cells in the MDSCs. CD10^+^ and CD10^–^ subsets are further gated in the PMN-MDSCs. (**b**) Inhibition of T cell proliferation in co-culture with high-density PMNs from healthy donor (hd-PMN normal, *n* = 10, grey triangles), high-density PMNs from patients (hd-PMN pt, *n* = 13, grey dots) or low-density PMNs subsets (CD10^+^, *n* = 11, black dots; CD10^−^, *n* = 10, black dots). Inhibition is shown as percentages of control T cell division index after a 4-days culture. Median and Inter-quartile-range (IQR). *p*-values of Wilcoxon matched pair-tests are indicated on top of pairs: *, *p* < 0.05; **, *p* < 0.01.

The capacity of low and high-density PMNs to suppress T cell proliferation was assessed using cells obtained from 28 metastatic melanoma patients and 10 healthy donors (Figure 1). Low-density PMNs were sorted from patients PBMCs depleted of CD3^+^ T cells as CD10^+^ or CD10^−^ subsets by FACS (Figure 1a). High-density PMNs were purified as described. Autologous T cells were used in 4-days proliferation assays (Figure 1b).

Low-density CD10^+^ PMNs significantly inhibited 32% of T cell proliferation as compared to the control, while low-density CD10^−^ PMNs significantly increased T cell proliferation by 20% (Figure 1b and Supplementary Figure S1). However, high-density PMNs were as suppressive as low-density CD10^+^ PMNs (mean inhibition of 36% and 32%, respectively). Although the range of inhibition was similar to the results of previous reports [28,29], the similar suppression of T cell proliferation by high-density PMNs from patients and from healthy donors was puzzling and questioned the specificity of the effect of PMN-MDSCs. We reasoned that the suppressive activity of PMN-MDSCs on T cell proliferation could be under-estimated considering the short ex vivo life span of high and low-density PMNs in co-culture with T cells (about 24 h, Supplementary Figure S2) and the length of the assay (4 days).

### 2.2. PMN-MDSCs Suppress T Cells by Direct Cytotoxicity

In pilot experiments, we assessed the viability of high-density PMNs, PMN-MDSCs and T cells over the course of the 4-day assay. High-density PMNs were isolated from a patient’ blood, PMN-MDSCs were isolated from the same patient’ PBMCs with CD15 magnetic beads and allogeneic T cells were isolated from healthy donor were used for co-cultures (Supplementary Figure S3).

**Figure 2:**
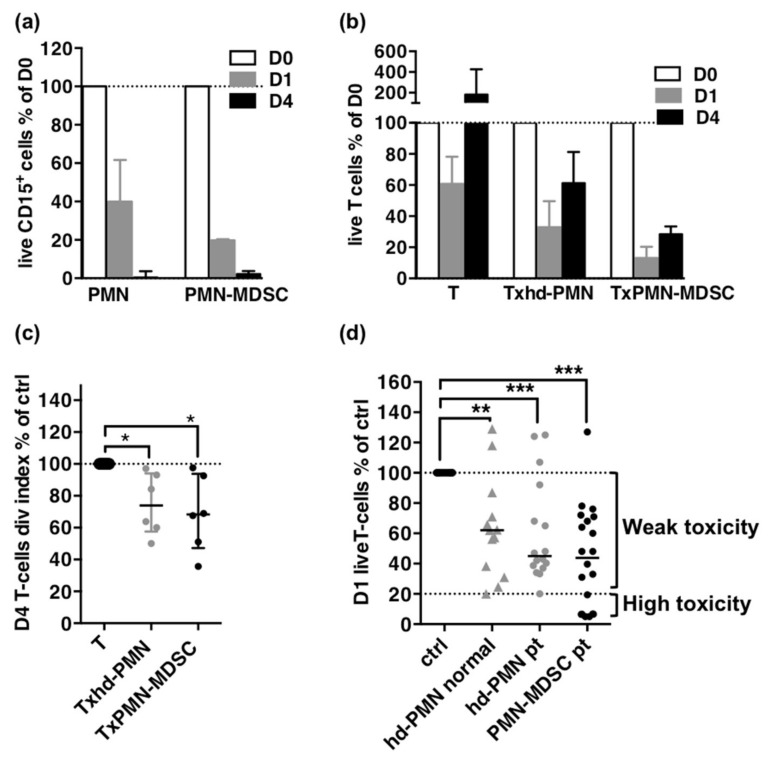
Suppression as direct cytotoxicity on T cells. (**a**) Follow-up of CD15^+^ cells during the proliferation assay. Percentages of day 0 absolute counts of live CD15^+^ cells on day 1 and day 4. (**b**) Follow-up of T cells during the proliferation assay. Percentages of day 0 absolute counts of T cells on day 1 and day 4. (**c**) Proliferation of T cells on day 4 as percentage of control T cells division index. For (**a**–**c**): high-density PMNs, *n* = 6; PMN-MDSCs, *n* = 5. (**d**). Cytotoxicity of high-density PMNs from healthy donor (hd-PMN HD, *n* = 13) or patients (hd-PMN pt, *n* = 16) and of PMN-MDSCs (*n* = 20) as percentages of control T cells survival in overnight co-cultures. The minimum value reached by high-density PMNs (20%) indicated by a dotted line constitute the threshold between weak and high cytotoxicity for PMN-MDSCs. Medians and IQR for (**a**–**c**); median for (**d**). *p*-values of Mann-Whitney *U*-tests for (**a**,**b**) and of Wilcoxon matched pairs-tests for (**c**,**d**) are indicated on top of pairs: *, *p* < 0.05; **, *p* < 0.01; *** *p* < 0.001.

At day 1, a mean of 57% of high-density PMNs and 87% of PMN-MDSCs were lost in the co-cultures with T cells and more than 98% of both had disappeared at day 4 (Figure 2a and Supplementary Figure S2a). T cells counts decreased by 40% in controls at day 1, as expected after thawing frozen samples, but in co-culture with high density PMNs or with PMN-MDSCs, the decrease reached 65% and 90% respectively suggesting a direct killing effect higher for PMN-MDSCs (Figure 2b). T proliferation, assessed by the division index, could not be detected during the first day of culture (Supplementary Figure S2c) and on day 4, was similarly decreased in co-culture with high-density PMNs or PMN-MDSCs by 25% and 32% respectively (Figure 2c and Supplementary Figure S2c).

These results suggest that cytotoxicity is also involved in the suppression of T cells and is a major difference between PMN-MDSCs and high density PMNs. Therefore we decided to evaluate the suppressive capacity of PMN-MDSCs in a cytotoxicity assay and expanded our experiments to a larger number of samples (Figure 2d).

PMN-MDSCs and high-density PMNs from patients or healthy donors displayed a significant cytotoxicity against T cells (Figure 2d). Despite a trend for PMN-MDSCs or high-density PMNs from patients to be more toxic than high-density PMNs from healthy donors (median survival of 44%, 45% and 62%, respectively), the differences were not significant. However, cytotoxicity was variable and seven out of the 20 PMN-MDSCs samples showed cytotoxicity below that of high-density PMNs, although similar ratios of effectors to targets were used (means of 0.7 and 0.8, respectively).

### 2.3. High Cytotoxicity Is Mediated by Interaction of PMN-MDSCs with T Cells through MAC-1

**Figure 3:**
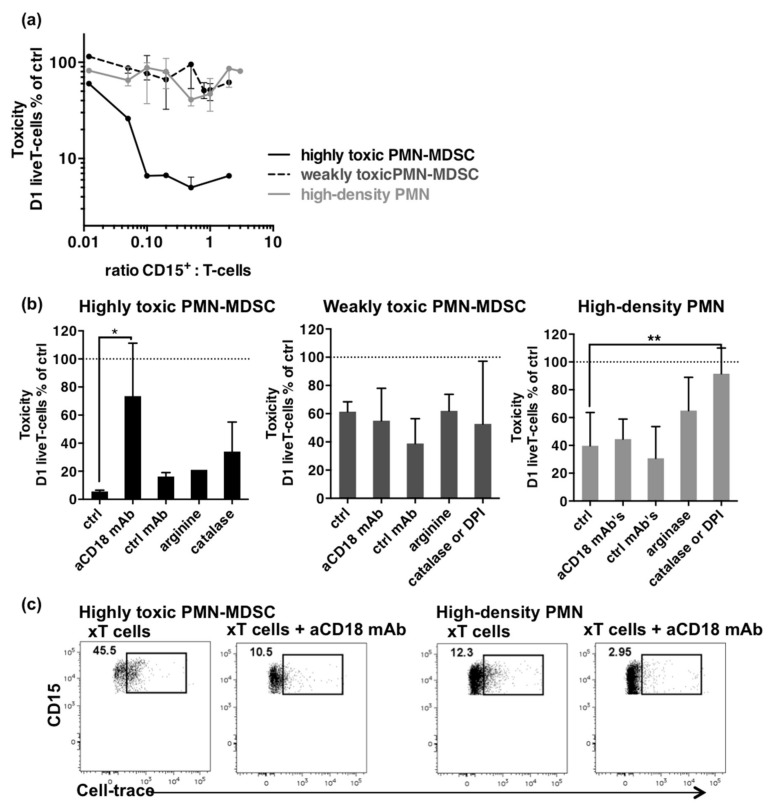
Mechanisms involved in cytotoxicity. (**a**) T cell survival according to CD15^+^ to T cell ratios of highly toxic PMN-MDSCs (black line), weakly toxic PMN-MDSCs (dashed line), and patients’ high-density PMNs (grey line). Data come from different experiments (highly toxic PMN-MDSCs, *n* = 6; weakly toxic PMN-MDSCs, *n* = 11; high-density PMNs, *n* = 16). Ratios were calculated from aliquots stained on day 0. Median and IQR. (**b**) Restoration of T cell survival in cytotoxicity assays by anti-CD18 mAb’s, control mAb’s (ctrl), arginine, and catalase or DPI. Highly toxic PMN-MDSCs, *n* = 4 for aCD18 and ctrl mAb’s; *n* = 1 for arginine; *n* = 2 for catalase. Weakly toxic PMN-MDSC, *n* = 10. High-density PMNs, *n* = 12. Median and IQR. *p*-values of Wilcoxon matched pair-tests are indicated on top of pairs: *, *p* < 0.05; **, *p* < 0.01. (**c**) Trogocytosis of T cells by PMN-MDSCs. Dot plots show expression of the Cell-trace T cells dye in highly toxic PMN-MDSCs and high-density PMNs after overnight co-cultures without or with anti-CD18 mAbs. PMNs were gated as live CD3^−^CD15^+^ cells.

When sufficient numbers of PMN-MDSCs were recovered, different ratios of effector to target were assessed in cytotoxicity assay. Samples were retrospectively divided into highly or weakly cytotoxic samples according to the threshold defined above (T cells survival of 20%). Ratio-effect curves were next plotted to evaluate whether cell-to-cell interactions mediated cytotoxicity (Figure 3a). Weakly toxic PMN-MDSCs and high-density PMNs showed no increase in toxicity against T cells, whereas the ratio-dependent cytotoxicity observed for the highly toxic PMN-MDSCs suggested a specific underlying mechanism.

Such strong cytotoxicity was previously described in PMNs subsets found in patients treated with LPS [32] or G-CSF [31]. Mechanisms that mediated the PMNs’ cytotoxicity involved interactions with T cells through MAC-1 [32] and the release of ROS or arginase-1 [31]. We tested whether similar mechanisms were driving cytotoxicity by performing restoration experiments with monoclonal antibodies (mAbs) against CD18 to inhibit interactions through MAC-1, arginine to compensate for depletion by arginase-1, catalase, a H_2_O_2_ scavenger, or diphenyleneiodonium (DPI, a NADPH oxidase inhibitor) to counteract ROS production.

High cytotoxicity was inhibited by anti-CD18 mAbs, suggesting a crucial role of cell-to-cell interactions through MAC-1 (Figure 3b). Catalase showed a trend to restore T cell survival but DPI could not be assayed to confirm the role of ROS in the process. Arginine did not inhibit cytotoxicity suggesting that arginase-1 was not involved, coherently to the short incubation time. Weak cytotoxicity was not significantly inhibited by any of the conditions tested. In contrast, cytotoxicity by high-density PMNs was significantly inhibited by catalase or DPI, but not by anti-CD18 mAbs, suggesting the central role of respiratory burst in the cytotoxicity against T cells. Thus, the strong cytotoxicity of PMN-MDSCs results likely from a close interaction with T cells through MAC-1 whereas the weak cytotoxicity of high-density PMNs is rather based on release of ROS in the culture medium.

Since MAC-1 is necessary for trogocytosis we investigated whether this mechanism was involved in the cytotoxicity of PMN-MDSCs by analyzing the expression of the Cell-trace T cells dye in PMN-MDSCs after overnight co-culture (Figure 3c). A high expression of Cell-trace was found in highly toxic PMN-MDSCs as compared to high-density PMNs (45.5% vs. 12.3%, respectively) and this expression was inhibited by anti-CD18 mAbs. This result suggested that highly toxic PMN-MDSCs might kill T cells using trogocytosis.

### 2.4. Enrichment in a Subset Co-Expressing Intermediate Levels of CD10 and CD16 Correlates with High Cytotoxicity of PMN-MDSCs

**Figure 4:**
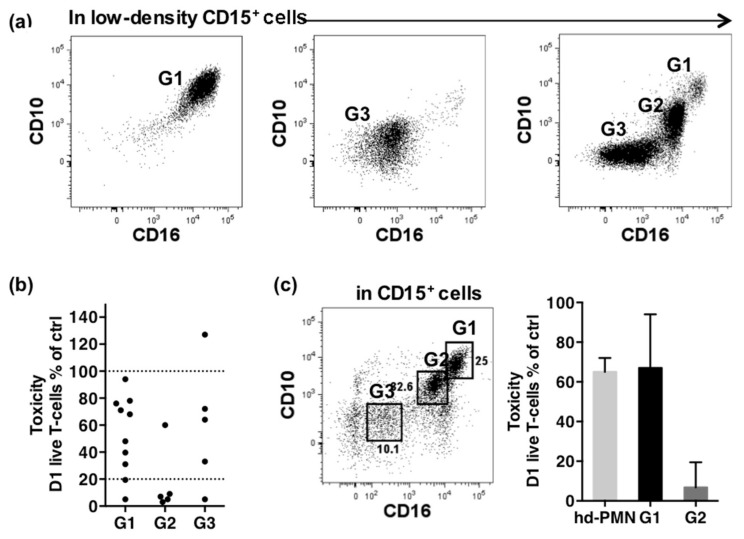
Matching of cytotoxicity with phenotype. (**a**) Typical CD10/CD16 profiles of PMN-MDSCs gated as CD14^−^CD15^+^ cells in PBMCs as described in Figure 1. The CD10^high^CD16^high^ population is named G1, the CD10^int^CD16^int^ population G2 and the CD10^−^CD16^−^ population G3. One patient’s sample is shown as an example for each typical profile observed. (**b**) Cytotoxicity according to the major subset present in CD15^+^ cells. 100% and the threshold of 20% are shown as dotted lines. Samples grouped as G1 contained on average 84% of G1, 2% of G2 and 6.8% of G3 (*n* = 10). Samples grouped as G2 contained on average 13% of G1, 48% of G2 and 29% of G3 (*n* = 5). Samples grouped as G3 contained on average 6% of G1, 14% of G2 and 53% of G3 (*n* = 5). (**c**) Cytotoxicity of G1 and G2 cells sorted by FACS from the PBMCs of a patient sample. Cytotoxicity of high-density PMNs of the same patient is shown (for each cell-type, 1 sample in triplicate).

We searched to identify the highly toxic PMN-MDSCs. As shown in Figure 1, PMN-MDSCs were heterogeneous according to the co-expression of CD10 and CD16, two markers acquired late during neutrophil differentiation [33]. Among the 20 samples analyzed, three typical profiles of PMN-MDSCs were found, suggesting three maturity levels (Figure 4a). The first (G1) expressed high levels of CD10 and CD16, typical of mature cells. The second and third, G2 and G3, expressed intermediate and lower levels of both markers respectively, suggesting more immature cells.

We associated phenotype to cytotoxicity by comparing samples according to the major PMN-MDSC subset they contained (Figure 4b). Four of seven highly toxic samples contained in majority G2 cells (more than 48%) while 8 of 12 weakly toxic samples contained in majority G1 cells (more than 84%). Samples enriched in G3 cells (more than 53%) showed various toxic profiles: one sample promoted T cell survival, 3 were weakly toxic, and one sample was highly toxic. Although imperfect, the matching strongly suggested that G2 cells were responsible for the high cytotoxicity. This conclusion was confirmed by comparing the cytotoxicity of G1 and G2 subsets sorted by FACS from a patient sample (Figure 4c). As expected, G1 showed weak cytotoxicity similar to high-density PMNs (T cell survival of 73% and 68% respectively), while G2 displayed strong cytotoxicity (T cell survival of 10%).

### 2.5. Phenotypic Characterization of the PMN-MDSCs Subsets

**Figure 5:**
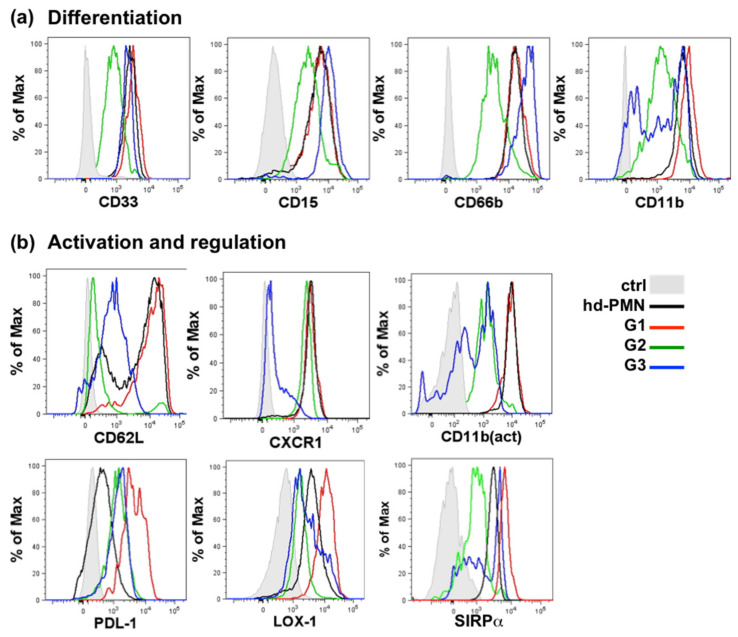
Phenotypic characterization of the G1, G2 and G3 subsets of PMN-MDSCs. Expression of markers sequentially expressed during granulocytic differentiation (**a**) and of markers of activation or regulation (**b**). Histograms show overlays of G1, G2, G3, high-density PMNs and control. An example of a typical expression profile was selected from 5 to 15 patient sample. For (**a**) control is unstained cells, for (**b**) control is matched isotype.

We characterized further the PMN-MDSCs subsets by analyzing the expression of surface markers typical for differentiation in addition to CD10 and CD16 [33] and markers of activation and regulation (Figure 5 shows histograms of a typical sample, Supplementary Figure S4 shows statistical analysis). The phenotype of the PMN-MDSC subsets was compared to the phenotype of high-density PMNs.

The expression profile for most surface markers was superimposable between G1 and high-density PMNs. The activated form of CD11b was the highest on both populations, but PD-L1 was expressed more highly on G1 and LOX-1 more frequently on G1 however with a large variability (Supplementary Figure S4b).

G2 ambiguously expressed weakly all markers of PMNs and especially markers of both early (CD66b) and late (CD11b) differentiation. The absence of CD62L and the expression of CXCR1 suggested that G2 were activated cells, although the activated form of CD11b, as well as PD-L1 and LOX-1 were weak. Interestingly, G2 expressed low levels of SIRPα.

G3 expressed the highest level of early differentiation marker CD66b and low levels of markers of activation (CD62L, CXCR1, PDL-1 and LOX-1) consistent with immature cells. However, CD11b, CXCR1 and SIRPα were bi-modal and variable in different samples, suggesting that this subset might contain different sub-populations that are indistinguishable by the expression of CD10 and CD16.

### 2.6. PMN-MDSC Subsets Are Differently Associated to Response to ICI Therapy

To evaluate the potential of PMN-MDSC subsets as bio-markers, we analyzed their frequencies in a cohort of 36 metastatic melanoma patients treated with ICI and 8 healthy donors (HD).

The population included 11 males and 25 females, median age 66 years (min 31-max 86). Thirteen patients were BRAF-mutated, seven patients were N-RAs mutated and 16 were BRAF and NRAS wild type. Immunotherapy was given as 1st line of systemic therapy in 28 patients, 2nd line in seven patients (previous treatment with BRAF-MEK inhibitors (*n* = 5), ipilimumab + nivolumab (*n* = 1), T-VEC (*n* = 1), and 3rd line in 1 patient (previous treatment with dacarbazine and ipilimumab). Fifteen patients received a combination of immunotherapy associating nivolumab and ipilumumab, one patient received a combination of pembrolizumab + anti IDO and 20 patients received anti-PD1 monotherapy.

Blood samples were taken at therapy start and during treatment for 20 patients, only at therapy start for seven patients and only during ICI for nine patients. Among the 36 patients, 13 patients achieved a complete response (CR) as best radiological response, nine patients were partial responders (PR), one patient achieved a stable disease (SD) and 13 patients had progressive disease (PD).

**Figure 6:**
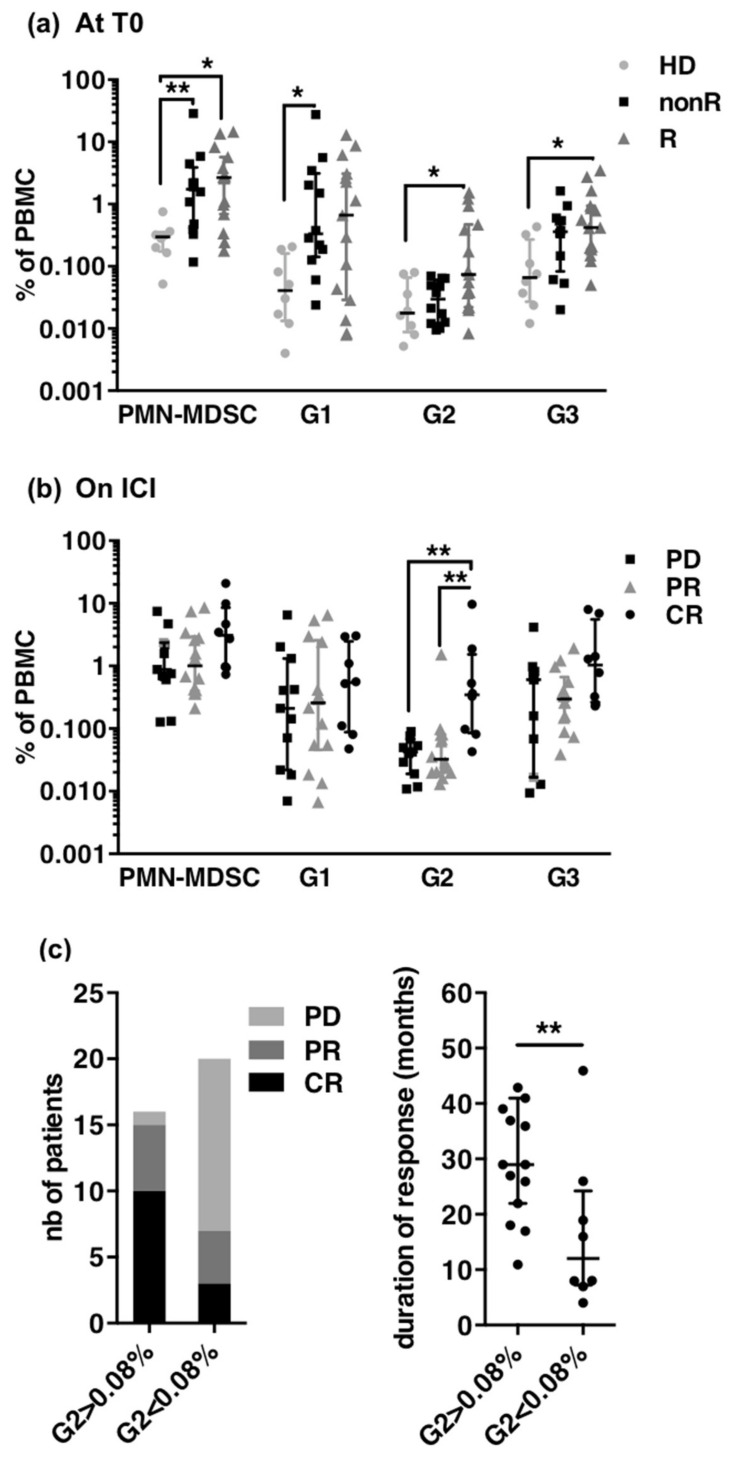
Frequencies of PMN-MDSC subsets in melanoma patients treated with ICI. (**a**) Frequencies of PMN-MDSCs, G1, G2 and G3 in PBMCs of HD (healthy donors, *n* = 8) and patients at treatment start according to best response achieved, non R (non responders, *n* = 12; 11 PD, 1 SD), R (responders, *n* = 15; 6 PR, 9 CR). Median and IQR. *p*-values of Dunn’s multiple comparison post Kruskall–Wallis test are indicated on top of pairs: *p* < 0.05 *; *p* < 0.01 **. (**b**) Frequencies of PMN-MDSCs, G1, G2 and G3 in patients according to the radiological response at the time of sampling (CR, *n* = 7; PR *n* = 11; PD *n* = 10; SD *n* = 1). Median and IQR, *p*-values of Dunn’s multiple comparison post Kruskall-Wallis test are indicated on top of pairs: *p* < 0.05 *; *p* < 0.01 **. (**c**) Radiological response (left) and mean duration of response (right) in patients according to G2 frequency above (*n* = 16, including 10 CR and 5 PR) or below (*n* = 20, including 3 CR and 4 PR responders) the threshold of 0.08%. *p*-values of Mann-Whitney *U*-tests are indicated on top of pairs: *p* < 0.01 **.

To investigated whether G1, G2 and G3 frequencies at treatment start were predictive of response to ICI, we first analyzed the blood of 27 patients sampled at treatment start and divided the patients into responders (nine CR and six PR) and non-responders (11 PD and one SD) (Figure 6a). As expected, patients showed increased frequencies of PMN-MDSCs as compared to healthy donors but irrespectively of the subsequent response to ICI (means of 4.4%, 4.1% and 0.3% for non-responders, responders and HD, respectively). G1 cells constituted the major subset of PMN-MDSCs in non-R (3.7%). G2 cells were significantly present only in responders (means 0.26% in responders vs. 0.035% in non-responders and 0.031% in HD, respectively). G3 cells were also significantly increased in responders (means of 0.85% in responders vs. 0.46 in non-responders and 0.13% in HD, respectively).

We next analyzed the blood of 29 patients sampled during ICI treatment. Since some patients had not reached their best radiological response at the time of sampling (five CR showed only a PR and two PR had still a PD), the closest RECIST radiological evaluation was used to classify patients. Nineteen patients were responders (seven CR and 11 PR) and 11 were non-responders (one SD and 10 PD) (Figure 6b). Frequencies of PMN-MDSCs and of G1 were not significantly different between the three groups while frequencies of G2 were significantly increased in complete responders as compared to PR and PD, and frequencies of G3 tended to be higher in CR.

Thus, high frequencies of the mature subset of PMN-MDSC (G1) at treatment start identified patients who will later not respond to ICI and increase of immature subsets seemed to identify patients who will achieve a complete response to ICI. To confirm the potential of G2 as biomarker, we next analyzed radiological response and duration of response according to G2 frequencies irrespectively of the timing of blood sampling (i.e., before or under ICI) (Figure 6c). The 90% percentile of frequencies in non-responders (0.08%) was used as threshold (Supplementary Figure S3). Out of the 36 patients, 16 had G2 > 0.08% and 20 patients had G2 ≤ 0.08%). Among the 22 responders (13 CR and nine PR), 15 had G2 > 0.08% and seven had G2 below 0.08%. The median duration of response was significantly longer in responders when G2 was above 0.08% (median of 34 months vs 16 months). Thus having G2 above the threshold was clearly a characteristic of responders although some responders were below this threshold.

### 2.7. Absence of Expression of SIRPα Identifies the Full Subset of Immature PMN-MDSCs Expanded during Response to ICI

Although G3 was the more abundant immature subset in most patient samples, G2 was a more relevant biomarker that identified a part of responders to ICI. The bi-modal expression of several markers and the variability of functional effect on T cells were arguments to suspect that the G3 subset contained different populations. Among bi-modal markers, SIRPα appeared particularly interesting because of its participation to the “don’t eat me” axis that regulates function of myeloid cells [19]. G2 did not express SIRPα consistently with their high toxic potential (Figure 5). We speculated that the G3 cells not expressing SIRPα might be precursor of G2 and that the whole immature subsets of PMN-MDSCs associated to efficient response to ICI could be identified in the immature compartment (G2 + G3) by the absence of expression of SIRPα.

We tested this hypothesis on a new small cohort of 19 patients sampled during ICI. Fourteen patients were sampled once, five patients were sampled twice during ICI.

**Figure 7:**
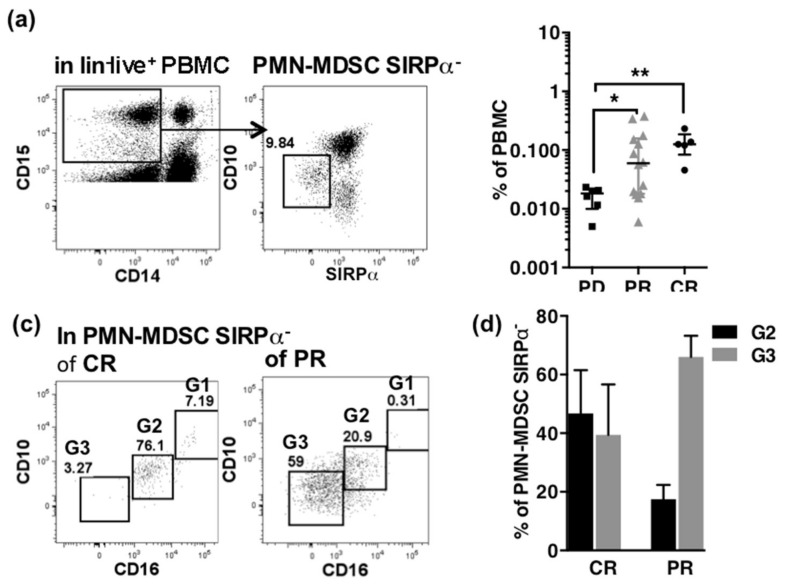
PMN-MDSC SIRPα^−^ as a biomarker of response to ICI. (**a**) Gating of PMN-MDSC SIRPα^−^ in lin**^-^**CD15^+^CD14^−^ PBMCs. (**b**) Frequencies of PMN-MDSC SIRPα^−^ according to the best radiological response to ICI (PD *n* = 6, CR *n* = 4, PR *n* = 9). *p*-values of Mann-Whitney *U*-tests are indicated on top of pairs: *p* < 0.05 *; *p* < 0.01 **. (**c**) Typical dot plots of co-expression of CD10 and CD16 in PMN-MDSC SIRPα^−^ of a patient with CR (left) and a patient with PR (right). Gates for G1, G2 and G3 are indicated. (**d**) Distribution of PMN-MDSC SIRPα**^−^** within G2 and G3 subsets according to best response to ICI (CR *n* = 5, PR *n* = 14).

The PMN-MDSC SIRPα**^−^** cells were gated in the lin**^−^**CD14**^−^**CD15**^+^** PBMCs (Figure 7a) and their frequencies were compared according to the best radiological response achieved (Figure 7b).

The population included 10 males and nine females, median age 68 years (min 20–max 79). Ten patients were BRAF-mutated, two patients were N-RAs mutated and seven were BRAF and NRAS wild type. Immunotherapy was given as 1st line of systemic therapy in 15 patients, 2nd line in three patients (previous treatment with BRAF-MEK inhibitors, and 4th line in one patient (previous treatment with dabrafenib + trametinib + anti-PD1/dabrafenib + trametinib/nivolumab). Nine patients received a combination of immunotherapy associating nivolumab and ipilumumab, two patients received a combination of nivolumab + relatlimab) and eight patients received anti-PD1 monotherapy.

Best radiological response achieved was PR in nine patients, CR in four patients and PD in six (including one SD) patients. Patients achieving a CR or PR showed a significant increase of low-density CD15^+^SIRPα^−^ cells as compared to PD (Figure 7b). Furthermore, the PMN-MDSC SIRPα^−^ cells were distributed in G2 and G3 subset in complete responders, while they were mainly of G3 phenotype in partial responders (Figure 7c,d).

## 3. Discussion

PMN-MDSCs are thought to support tumor progression and resistance to ICI therapy through their suppressive functions. Although high frequencies in the blood are known as a bad prognosis marker [6] and early decrease on ICI therapy correlates to response [7], PMN-MDSCs are not yet validated as biomarkers to monitor melanoma patient’s response to ICI. PMN-MDSCs contain both mature and immature cells and this heterogeneity, that has not been considered so far, may limit their use as biomarkers. We report here that while mature subsets inhibit T cell proliferation, immature subsets suppress T cells by direct cytotoxicity. Although both are by definition PMN-MDSCs, increased numbers of immature subsets correlate with response to ICI, challenging the concept that MDSC play an exclusively negative role in cancer.

PMN-MDSCs with high levels of CD10, CD16 and CD11b typical of an activated mature phenotype are the most frequent subset in metastatic melanoma patients. Consistently with previous observations showing that PMN-MDSCs correlate with poor prognosis in several types of cancers [6,7,34,35], we found that increased frequencies of PMN-MDSCs at the start of ICI in MM patients correlated with poor subsequent response to treatment suggesting a contribution to primary resistance to ICI therapy. Inhibition of T-cell proliferation is associated with cells that express CD10, a late marker of differentiation [28,31], or LOX-1, a class-E scavenger receptor [29,36]. We found that CD10^+^CD16^+^ PMN-MDSCs inhibited T cells proliferation and showed a weak but significant capacity to kill T cells directly however not significantly different from high-density PMNs from patients or healthy donors. Because release of ROS was the mechanism underlying the cytotoxicity of high-density PMNs, it is conceivable that this cytotoxicity represents an artifact resulting from the triggering of respiratory burst during the preparation of PMNs, as suggested previously [26,37]. This artifact could hide the specific suppressive capacity of PMN-MDSCs. LOX-1 was not measured on the samples analyzed in this set of functional assays. However, the high variability of frequency of LOX-1 analyzed on an additional cohort of 16 metastatic patients suggests that activated PMNs might be more frequent than suppressive PMN-MDSCs in the low-density CD10^+^CD16^+^ cells of those patients and could explain the absence of high suppression.

Our result shows that immature cells constitute an important part of the PMN-MDSCs in some MM patients. The more frequent population lacks expression of CD10 and CD16 and expresses high level of CD66b typical of immature cells. However, the bi-modal expression of several surface markers (CD11b, CXCR1 and SIRPα) and the different functional effects (immune-stimulatory activity or neutrality) suggests overlap of different populations. Immune-stimulatory activity of CD10^−^ low-density PMNs was described in patients in whom inflammation is induced by G-CSF [31] and it is enticing to speculate that the contrary effects on T cell are directly associated to pro- or anti-tumoral functions [12,38]. We could not establish a correlation of the expansion of immature cells with response to ICI in our cohort of patients probably because immune-stimulatory cells could not be identified within the pool of immature cells.

A less frequent immature population characterized by an intermediate expression of CD10 and CD16 displayed a strong cytotoxicity against T cells by interacting with MAC-1 and was expanded in patients responding to ICI, either before or during treatment. Complete radiological response, and higher duration of response were observed in patients presenting increased levels of this immature subset.

MAC-1 is involved in many PMNs functions, including phagocytosis [39]. PMNs were recently shown to kill target cells opsonized with antibodies by trogoptosis [17]. Trogoptosis result from the lytic processes induced by trogocytosis, which consists in phagocytosis of the target cell membrane [16]. Highly toxic PMN-MDSCs displayed their capability to engulf T cell components in a MAC-1-dependent manner. Moreover, the absence of dead cells in the co-cultures is coherent with a necrotic death. Together, these results suggest that highly toxic PMN-MDSCs might use similar processes to kill T cells. Phagocytosis is controlled by the “don’t eat me” axis constituted by the interaction of the myeloid checkpoint SIRPα with CD47, a self-molecule widely expressed on healthy cells [19]. The absence of SIRPα on CD10^int^CD16^int^ PMN-MDSCs might be consistent with the high cytotoxicity of these cells, although activation or “eat me” signals are also required to induce phagocytosis. Further studies will be necessary to understand how these cells get activated, whether they enter the tumor and whether the cytotoxicity is specifically targeted to T cells or to the whole tumor environment.

Absence of SIRPα expression permitted to identify immature PMN-MDSCs expanded both in CR and PR. In complete responders, PMN-MDSCs SIRPα^−^ were distributed in the CD10^int^CD16^int^ and CD10^−^CD16^−^ subsets, while in partial responders they were mostly in the CD10^−^CD16^−^ subset. Given the nature of PMNs, it is tempting to speculate that CD10^int^CD16^int^cells derive from CD10^−^CD16^−^ cells by uncontrolled activation during their handling in vitro. The expression of SIRPα on the whole immature cells of non-responders demonstrates the reliability of this marker to identify the immature cells associated to response in the populations of different origin that overlaps in immature PMN-MDSCs.

Similar population of toxic PMNs were described in patients with lipopolysaccharide- (LPS) [32] or granulocyte colony-stimulating factor- (G-CSF) [31] induced inflammation but not, to our knowledge, in cancer. This population was proposed to be a separate subset of neutrophils recruited in the blood during inflammation [40]. Our results suggest an important role for PMNs in the inflammatory events associated to efficient ICI therapy [41]. The expansion of a subset of PMNs devoid of regulating pathways that results in the suppression of healthy T cells during ICI, a therapy that precisely aims to restore anti-tumor T cell immunity, is rather paradoxical and highlights the limits of our understanding of the processes that lead to tumor reduction [41]. When and where this function could take place in vivo remains to be investigated. In this work cytotoxicity has been a tool to speculate that absence of SIRPα could be a convenient marker to monitor those cells. The validation of SIRPα^−^ PMN-MDSCs as biomarkers of response to ICI therapy on larger number of patients is warranted and would further strengthen our understanding of this cellular compartment.

## 4. Materials and Methods

### 4.1. Human Blood Samples

Blood samples were obtained from patients treated with ICI for a metastatic melanoma between December 2015 and September 2020 sampled at different time points i.e., before or during ICI therapy. According to clinical recommendations, ICI was interrupted in patients for whom a complete response was achieved after 2 years of therapy or earlier in case of toxicity.

Samples from 28 patients were used only for functional analysis. They included 19 males and nine females, median age 68 years (min 37–max 80). Nine patients were BRAF-mutated four patients were N-RAS mutated and 16 were BRAF and NRAS wild type. Immunotherapy was given as 1st line of systemic therapy in 19 patients and ≥2 line in nine patients. Ten patients received a combination of immunotherapy associating nivolumab and ipilumumab, two patients received a combination of nivolumab + anti IDO) and 16 patients received anti-PD1 monotherapy.

Samples from 36 patients were used to investigate correlations of G1, G2 and G3 subsets with radiological response. Samples from 19 additional patients were used for the study of SIRPα Detailed description of these populations are provided in the results session. Healthy donor’s samples were obtained from healthy volunteers (Etablissement français du sang, EFS, Marseille, France).

### 4.2. Radiological Response Assessment

According to clinical practice, full body CT-scans were performed every 3-months and RECIST 1.1 criteria were used to assess radiological response to ICI.

### 4.3. Cell Preparation

Isolation of PBMCs was performed between 4 and 18 h after blood collection by centrifugation on Ficoll Hypaque gradients (MSL, density 1.077 g/L, Eurobio, Paris, France) for 20 min at 850 g. PBMCs were taken at the interface between the plasma and Ficoll and washed twice at low speed. PMNs contained within this low-density fraction were named PMN-MDSCs.

Two procedures were used to separate PMN-MDSCs from PBMCs. In a first series of experiments shown in Figure 1, low-density PMNs were enriched by depletion of CD3^+^ cells with magnetic beads (Miltenyi, Bergisch Gladbach, Germany), stained with Panel 1 antibodies and sorted by FACS (ARIA III, Becton Dickinson, Le Pont-de-Claix, France) as CD3^−^CD56^−^CD19^−^ (lin^−^) CD33^+^HLA-DR^lo^CD15^+^CD14^−^CD10^+^ or lin^–^CD33^+^HLA-DR^lo^ CD14**^-^**CD15^+^CD10^−^ (gating strategy shown in Figure 1). For results shown in Figure 2, Figure 3 and Figure 4, PMN-MDSCs were sorted from PBMCs with CD15 magnetic beads. The purity of cells preparations was usually above 85% (shown in Supplementary Figure S2).

High-density PMNs were taken at the interface between Ficoll and red blood cells (RBCs). RBCs were lysed using RBC lysing buffer (Thermo Fisher, Illkirch, France). PBMCs, PMN-MDSCs and PMNs were re-suspended in RPMI medium supplemented with 10% inactivated fetal calf serum and antibiotics. 

Autologous T cells collected by depletion of CD3^+^ cells from PBMCs of patients in the study or T cells sorted with CD3 magnetic beads from thawed allogeneic PBMCs from healthy donors were used in suppression assays as indicated. T cells were stained with 5 µM CellTrace (Life Technologies, Villebon-sur-Yvette, France) and re-suspended in RPMI medium supplemented with fetal calf serum and antibiotics.

### 4.4. Inhibition of T Cells Proliferation

CD3-CD28 coated beads (Dynabeads) were used to stimulate T cells at a ratio of 1:5 T cells and culture medium was supplemented with 100 ng/mL IL-2 (Thermo Fisher, Illkirch, France). Co-cultures with PMN-MDSCs or with high-density PMNs were set at a ratio of 1:1. 50,000 T cells were used per condition. Control consisted of T cells cultivated alone. Samples were stained with Panel 2 and analyzed by FACS at day 0 to check purity and ratio, and at day 4 to analyze proliferation by Cell-trace dilution. Gates were set on live Cell-trace^+^ cells and the proliferation was quantified by the division index calculated with the FlowJo tool (Treestar, Ashland, OR, USA) (see Supplementary Figure S2c). Results were expressed as percentages of control T cells division index. No proliferation could be detected on day 1 (Supplementary Figure S2c).

### 4.5. Cytotoxicity Assay

Counting beads (BD Biosciences, Le Pont-de-Claix, France) were added to the Cell-trace stained T cell suspensions. Co-cultures with PMN-MDSCs or with high-density PMNs were set at ratios of 1:10 to 2:1. 50,000 T cells were used per condition. Control consisted of T cells cultivated alone. Samples were stained at day 0 and on day 1 with Panel 2 and analyzed by FACS. Gates were set on counting beads, on live CD3^+^ Cell-trace^+^ cells and on live CD15^+^ cells (see Supplementary Figure S2a). Day 0 staining permitted to check the purity of the cell sorting and the effective ratio of PMNs to T cells. Purity was routinely greater than 85% (Supplementary Figure S3). Cytotoxicity was evaluated on day1 staining, live Cell-trace^+^ T cells counts were standardized per counting bead and results were expressed as percentages of control T cells. Trogocytosis of T cells by PMNs was evaluated by FACS analysis of the expression of Cell-trace dye in PMNs, gated as CD3^−^CD15^+^ cells after overnight co-culture with T cells.

### 4.6. Inhibition of Cytotoxicity

To inhibit suppression, catalase at 50 µg mL^−1^, DPI (diphenyleneiodonium) at 5 µM and arginine at 1 µM were used (all products from Sigma-Aldrich, Merck, Saint-Quentin-Fallavier, France). Anti-CD18 monoclonal antibody comes from an in-house ascitic fluid appropriately diluted and was controlled by an ascitic fluid of an in-house anti-human HLA-ClII mAb.

### 4.7. Flow Cytometry

Panel 1 included antibodies allowing the gating of PMN-MDSCs in the PBMCs and the analysis of expression of surface markers. It comprised of CD3-BV510, CD56-BV510, CD19-BV510, CD11b-APCH7, CD15-BV711, CD14-BV650, HLA-DR-BV605, CD10-PE-CF594 (Becton Dickinson), CD16-AF700 (BioLegend, Ozyme, Saint-Cyr-l’Ecole, France), CD33-PC7 (eBiosciences, Thermo Fisher), LIVE/DEAD fixable aqua (Life Technologies, Villebon-sur-Yvette, France). LOX-1-PE, CD11b-activated-PE (clone CBRM1/5) (Bio-Legend, Ozyme), CD62L-PE, CD66b-PE, PDL-1-BV421, CXCR1-PE, CD172ab-AF647 (BD Biosciences) were alternately included in Panel 1.

Panel 2 was used for functional analysis. It contained CD15-FITC (eBiosciences, Thermo Fisher), CD3-PC5 (BD Biosciences) and LIVE/DEAD near-IR (Live Technologies,). CD66b-PE, CD10-PE-CF594 and CD16-AF700 were occasionally added. PBMCs were stained 20 min at room temperature with fluorescent reagents pre-mixed in PBS, washed, then fixed with 4% paraformaldehyde and analyzed by FACS within 4 days on LSRII-SORP cytometer (BD Biosciences) equipped with four lasers (405 nm/100 mW, 488 nm/100 mW, 560 nm/50 mW and 630 nm/40 mW). PMT were set using unstained and fully stained samples. Compensations were performed with beads stained with corresponding reagents. Data were exported and analyzed with FlowJo (version 9-2, MacOS X).

### 4.8. Statistics

Statistical graphics were performed with Prism 6 software (Ritme, Paris, France). Mann–Whitney *U*-test, Wilcoxon matched pair-test, Kruskall–Wallis followed by Dunn’s post-hoc test were used as indicated.

## 5. Conclusions

Taken as a whole, PMN-MDSCs are known as biomarkers of poor prognosis in cancer. They are constituted in majority of mature cells that can hide discrete immature populations associated with the acute inflammation induced by the response to immunotherapy. These immature cells are characterized by the absence of expression of SIRPα, a marker deduced from functional studies, and are promising biomarkers for monitoring patients under ICI, although further studies on larger cohorts will be necessary for validation. Although the expansion of these populations during the acute inflammation induced by immunotherapies is understandable, their immunosuppressive capacities seem paradoxical and show that much remains to be known about the role of PMNs in the immune response.

## Data Availability

The data presented in this study are available on request from the corresponding author. The data are not publicly available because most of the data correspond to patents’ data and the novel French laws (RGPD) include major limitations for their distribution.

## Supplementary Materials

The following are available online at https://www.mdpi.com/2072-6694/13/6/1362/s1. Supplementary Figure S1. Inhibition of proliferation of T cells by PMN-MDSCs and high-density PMNs. Examples of proliferation of T cells in co-culture with high-density PMNs from patient (pt) or from healthy donor (normal) and low-density PMNs CD10^+^ and CD10-from pt on day 4. Proliferation of matched T cells control is shown on the left. Division index are displayed in each graph. Supplementary Figure S2. Follow-up of cells survival in proliferation assays. (a) Gating strategy of cells in co-culture shown on the co-culture of T cells x high-density PMNs. (b) Viability staining of T cells (left panel) and of PMNs or PMN-MDSCs (right panel) in co-cultures. Top line shows day 0, bottom line shows day 1. Absolute counts of live cells standardized by counting beads are shown in graphs. For day 1 absolute counts of dead cells are shown in parentheses. (c) Proliferation of T cell. No proliferation is detected in control T cells on day 1. Proliferation of co-cultures is analyzed on day 4. Division index is displayed in the graphs. Supplementary Figure S3. Purity of cell preparations. Top line shows PMN-MDSCs after separation from PBMCs with CD15 magnetics beads. Bottom line shows high-density PMNs separated from blood after centrifugation on ficoll and lysis of red cells. Sequential dot plots shows FSC/SSC, SSC/viability, CD3/CD14 and CD66b/CD16 co-expression. Purity of cells preparations were respectively of 95% and 91%. A minor contamination of T cells but not of monocytes is observed. Supplementary Figure S4. Phenotypic characterization of the G1, G2 and G3 subsets of PMN-MDSCs. Expression of markers sequentially expressed during granulocytic differentiation (a) and of markers of activation or regulation (b). Histograms with bars show ratio of mean fluorescence intensity (MFI) to MFI of control. Histograms with scatter dots show percentage. *n* = 5 to 15 according to marker and subset. For (a), control is unstained cells, for (b) control is matched isotype. Median and IQR. p-values of Dunn’s multiple comparison post Kruskall–Wallis test are indicated on top of pairs: *, *p* < 0.05; **, *p* < 0.01; ***, *p* < 0.001. Supplementary Figure S5. Threshold for G2 is defined as the 90% percentile of the distribution of the frequencies in non responders at T0 and on ICI.
