# Supplementary material for: Identification of an Immature Subset of PMN-MDSC Correlated to Response to Checkpoint Inhibitor Therapy in Patients with Metastatic Melanoma
Source: Cancers (Basel). 2021 Mar 17;13(6):1362. doi: 10.3390/cancers13061362 (PMC8002694; doi:10.3390/cancers13061362)
Supplement: Supplementary file 1 [file cancers-13-01362-s001.pdf]

Supplementary figure 1

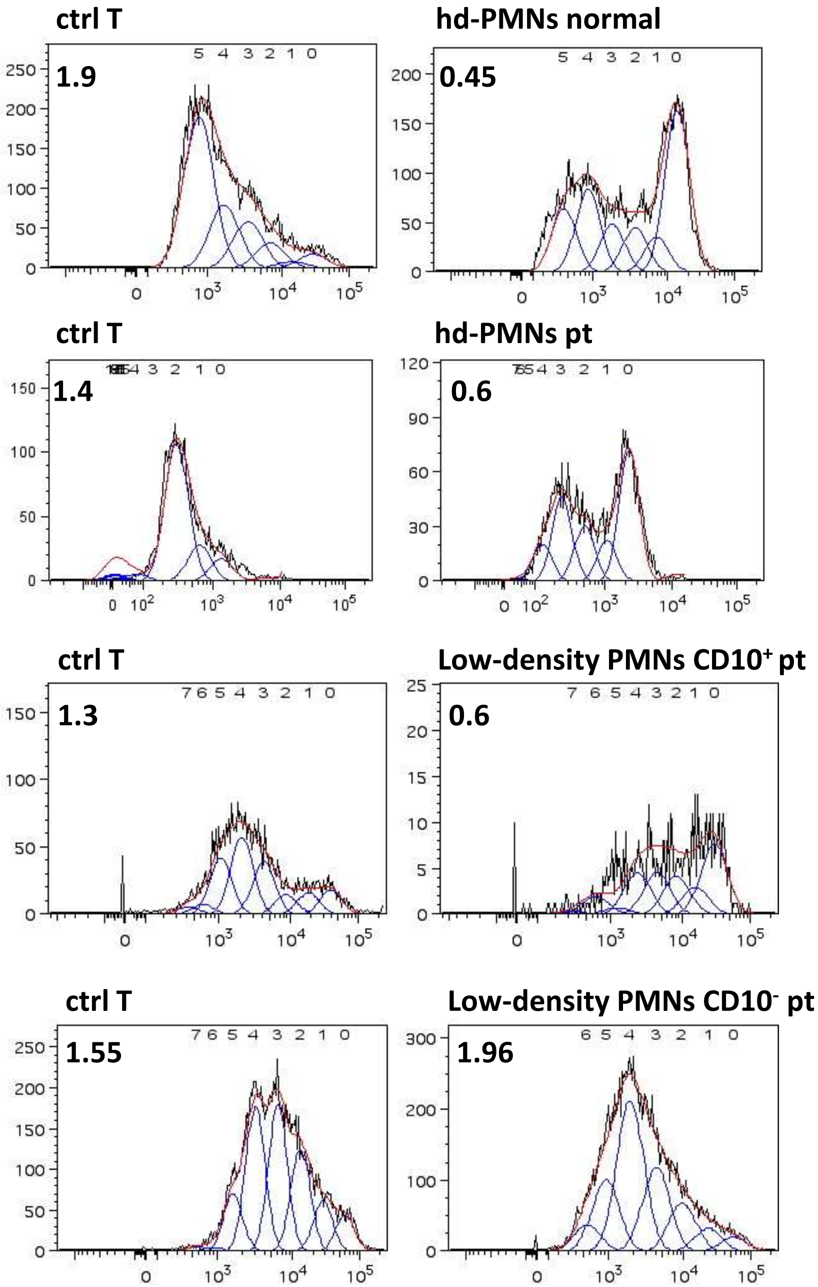

Supplementary figure 1. Inhibition of proliferation of T cells by PMN-MDSCs and high-density PMNs. Examples of proliferation of T cells in co-culture with high-density PMNs from patient (pt) or from healthy donor (normal) and low-density PMNs $CD10^{+}$ and $CD10^{-}$ from pt on day 4. Proliferation of matched T cells control is shown on the left. Division index are displayed in each graph.

Supplementary figure 2

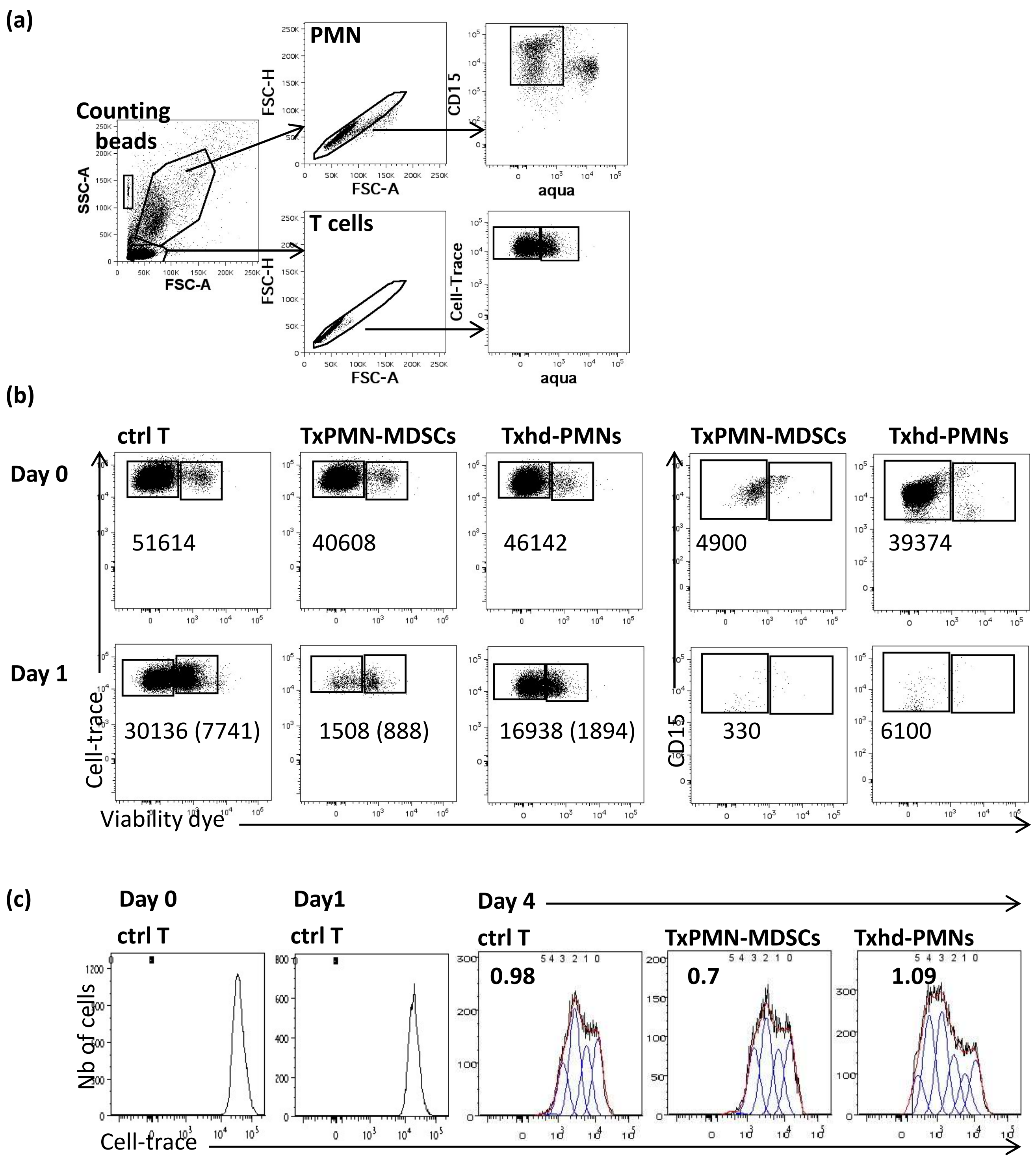

Supplementary figure 2. Follow-up of cells survival in proliferation assays. (a) Gating strategy of cells in co-culture shown on the co-culture of T cells x hd-PMNs. (b) Survival of cells in overnight co-cultures. Viability staining of T cells (left panel) and of PMNs or PMN-MDSCs (right panel). Top line shows day 0, bottom line shows day 1. Absolute counts of live cells standardized by counting beads are shown in graphs. For day 1 absolute counts of dead cells are shown in parentheses. (c) Proliferation of T cell. No proliferation is detected in control T cells on day 1. Proliferation of co-cultures is analyzed on day 4. Division index is displayed in the graphs.

Supplementary figure 3

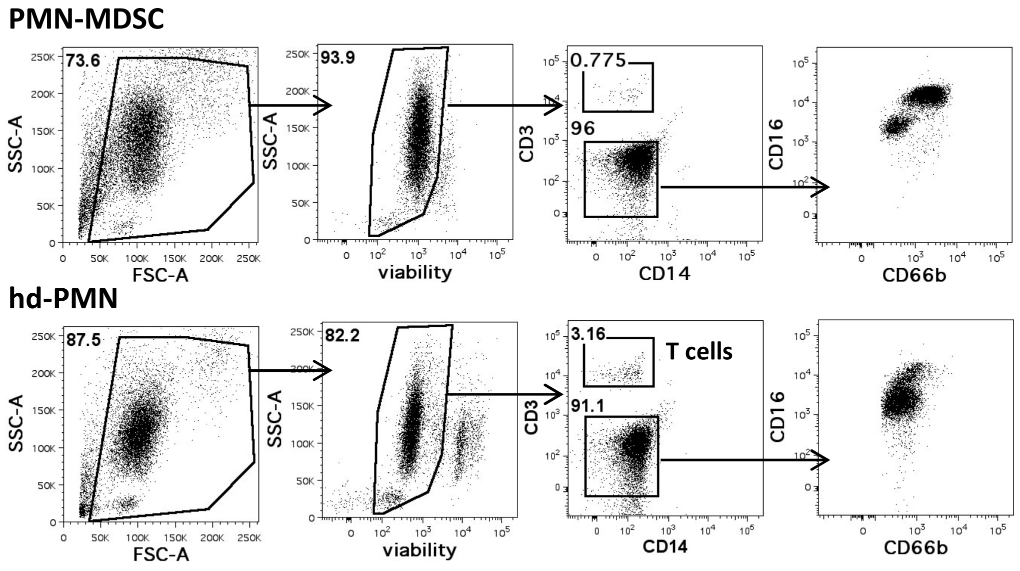

Supplementary figure 3. Purity of cell preparations. Top line shows PMN-MDSCs after separation from PBMCs with CD15 magnetics beads. Bottom line shows high-density PMNs separated from blood after centrifugation on ficoll and lysis of red cells. Sequential dot plots shows FSC/SSC, SSC/viability, CD3/CD14 and CD66b/CD16 co-expression. Purity of cells preparations were respectively of 95% and 91%. A minor contamination of T cells but not of monocytes is observed.

Supplementary figure 4

(a) Differentiation

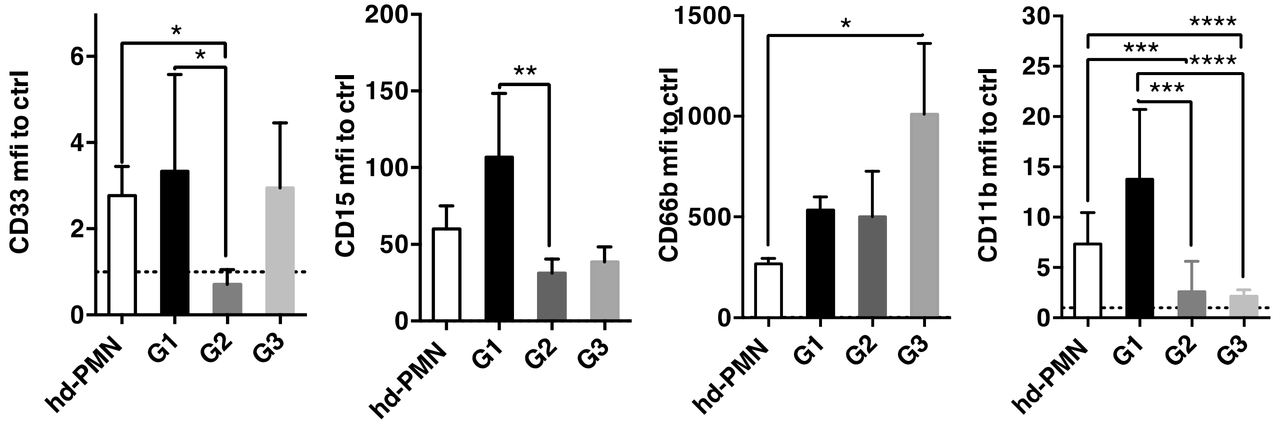

(b) Activation and regulation

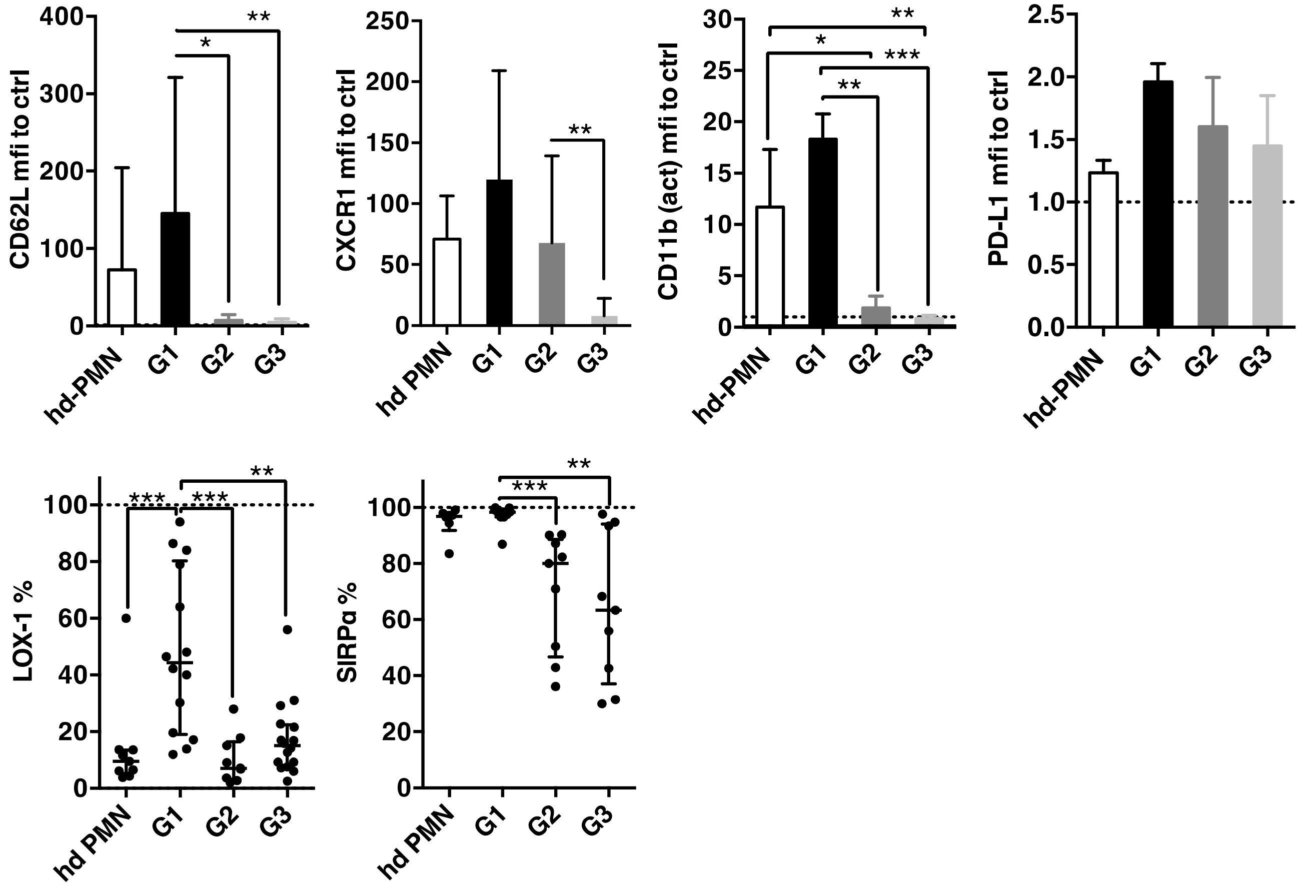

Supplementary figure 4. Phenotypic characterization of the G1, G2 and G3 subsets of PMN-MDSCs. Expression of markers sequentially expressed during granulocytic differentiation (a) and of markers of activation or regulation (b). Histograms with bars show ratio of mean fluorescence intensity (MFI) to MFI of control. Histograms with scatter dots show percentage. N=5 to 15 according to marker and subset. For (a), control is unstained cells, for (b) control is matched isotype. Median and IQR. *P*-values of Dunn's multiple comparison post Kruskall–Wallis test are indicated on top of pairs: *, $P < 0.05$; **, $P < 0.01$; P <0.001 ***.

Supplementary figure 5

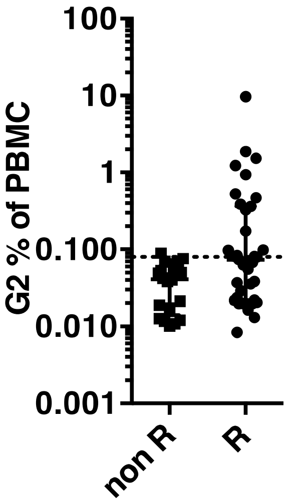

Supplementary figure 5. Threshold for G2 is defined as the 90% percentile of the distribution of the frequencies in non responders.
